# The disinformation playbook: how industry manipulates the science-policy process—and how to restore scientific integrity

**DOI:** 10.1057/s41271-021-00318-6

**Published:** 2021-11-22

**Authors:** Genna Reed, Yogi Hendlin, Anita Desikan, Taryn MacKinney, Emily Berman, Gretchen T. Goldman

**Affiliations:** 1 Union of Concerned Scientists, 1825 K Street NW, Ste 800, Washington, DC 20006 USA; 2 Dynamics of Inclusive Prosperity Initiative, Erasmus School of Philosophy, Erasmus University, Rotterdam, The Netherlands; 3 Environmental Health Initiative, University of California San Francisco, San Francisco, CA USA

**Keywords:** Disinformation, Scientific integrity, Science policy, Independent science, Regulatory capture, Conflicts of interest

## Abstract

For decades, corporate undermining of scientific consensus has eroded the scientific process worldwide. Guardrails for protecting science-informed processes, from peer review to regulatory decision making, have suffered sustained attacks, damaging public trust in the scientific enterprise and its aim to serve the public good. Government efforts to address corporate attacks have been inadequate. Researchers have cataloged corporate malfeasance that harms people’s health across diverse industries. Well-known cases, like the tobacco industry’s efforts to downplay the dangers of smoking, are representative of transnational industries, rather than unique. This contribution schematizes industry tactics to distort, delay, or distract the public from instituting measures that improve health—tactics that comprise the “disinformation playbook.” Using a United States policy lens, we outline steps the scientific community should take to shield science from corporate interference, through individual actions (by scientists, peer reviewers, and editors) and collective initiatives (by research institutions, grant organizations, professional associations, and regulatory agencies).

## Introduction

Science shapes our understanding of, and efforts to improve, people’s health, and well-being. If these efforts threaten an industry’s commercial interests, however, the industry worldwide may attempt to suppress or undermine the underlying science. Researchers have long chronicled the infamous example of the tobacco industry [1]. For decades, tobacco companies minimized the dangers of smoking, falsely presented low-tar and filtered cigarettes as safer, and denied the science demonstrating the hazards of secondhand smoke [1–3]. The success of the “tobacco playbook” made it a template for others, including the lead [4], sugar [5], and oil and gas industries [6].

While industry can target every step in the policymaking process, science—on which health-based government decisions depend—is especially vulnerable [3]. Science relies on constructive critique to spur research, ensure rigorous results, and test hypotheses. By reframing this procedural scrutiny as “doubt,” industry can undermine commercially inconvenient science. For industry, this means that debating the science is a shortcut to debating policy, making attacks on science a powerful tactic to shape regulation and insulate against litigation [2]. Understanding the disinformation playbook can help public health professionals, policymakers, and the public recognize and resist corporate interference in science.

As science has become increasingly politicized, it is more important than ever to analyze how scientific knowledge leads to public health policies that protect populations. The industry disinformation playbook enables corporate actors to undermine health-protective public policy, instead bending science to fit political ideologies at the expense of public health. This analysis presents case studies that illustrate this playbook’s dangers and offers policy mechanisms that can help prevent similar cases of corrupted science in the future. While this analysis focuses primarily on the United States, the problems and solutions described are applicable elsewhere, experienced especially in other high-income countries. As Europe and the US often serve as policy benchmarks for other countries, it is doubly important that rich democratic countries set a positive precedent for dealing with the runaway power of industry and its efforts to deprioritize public health.

## Disinformation playbook tactics

**Fig. 1:**
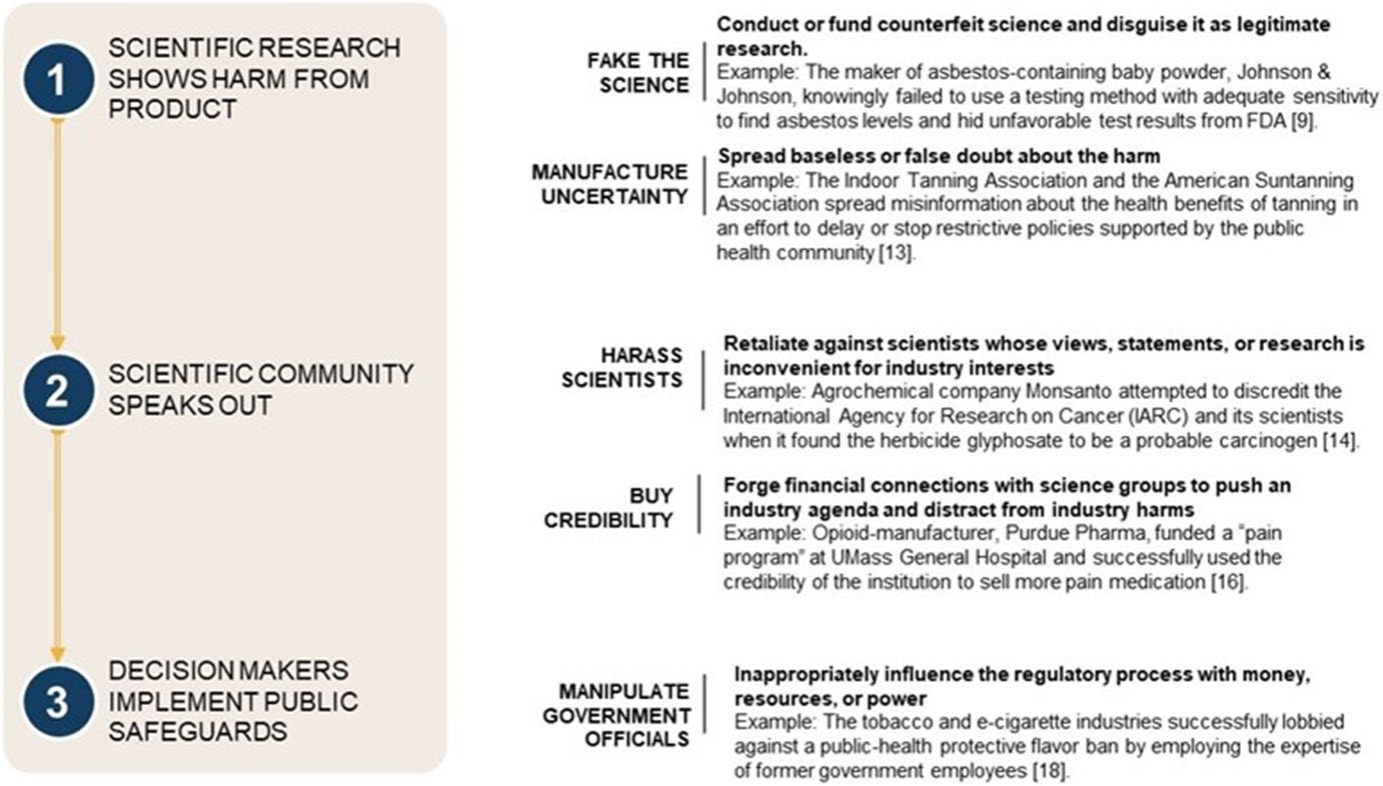
Five tactics used by industry to undermine science. The disinformation playbook tactics are employed by industry during the scientific process and the science-based decision-making process

While there are many tactics used by industry to obscure science [7], we focus on five tactics that most directly affect the science-policy interface (Fig. 1). Some “plays” occur internally—companies may conduct biased studies, use or publicize only favorable results, suppress unfavorable results, and retaliate against scientists. Others involve external stakeholders, such as government scientists and elected officials.

## Faking science: conducting—or paying others to conduct—flawed or biased scientific studies, or hiding research with unfavorable conclusions

Industry-sponsored research is more likely to have favorable outcomes for the target product or process than research funded by other sources, a phenomenon known as the “funding effect” [8]. Companies can:

- Publish studies with flawed methodologies (such as overlooking cumulative exposure), or bury studies with unfavorable results;
- Fund university scientists with explicit or implicit strings attached (for example, reserving the right to edit results);
- Hire firms from the “product defense industry” to deliver studies skewing the science in favor of a company’s agenda [2];
- Hide conflicts of interest for industry-funded or -affiliated scientists who publish papers, give testimony, or comment publicly;
- Publish ghostwritten articles in journals or media outlets; or
- Interfere with studies during the prepublication process, including peer review.

An example of “faking the science” was borne out by the company Johnson & Johnson (J&J). Company officials knew as early as 1971 that its talcum baby powder was contaminated with tremolite, an asbestos fiber and known carcinogen. They knew the levels at which tremolite could be detected and, as evidenced by internal documents, chose not to issue a testing method with better sensitivity and actively fought scientist and regulator attempts to do so. J&J’s failure to report at least 12 independent tests conducted over a 15-year period that found asbestos in its product meant that the United States (US) Food and Drug Administration (FDA) did not have adequate scientific information to make a regulatory decision. There is evidence that, in 1972, J&J manipulated the findings of one of the tests sent to the FDA by deleting the total tremolite content found in its baby powder product. FDA’s susceptibility to industry pressure, coupled with insufficient or biased company-submitted data, means that proper scrutiny of these products has been delayed. Over 19,000 lawsuits are currently pending against J&J related to harms caused by its powders, including ovarian cancer [9]. J&J took the powder off the market in North America in 2020 [10] but still affirms its safety.

## Manufacturing uncertainty: questioning credibility, or emphasizing uncertainty, of independent science unfavorable to industry interests

Companies can overemphasize scientific uncertainty through public relations campaigns, features in media outlets, political lobbying, or comments in regulatory dockets or congressional testimony. Often, companies target a single offending study for undermining their objectives. Alternatively, they may criticize an entire field, like epidemiology, for not making confidential data accessible to the public. This restricts the evidence allowed in policy decisions. Such maneuvers turn principles of transparency against science [11]. Companies also shield themselves from direct scrutiny by working through public relations firms, trade associations, or scientists they employ.

For example, strong evidence demonstrates the risks of tanning [12], but the indoor tanning industry undermines this consensus by overemphasizing the importance of vitamin D and questioning links between UV exposure and skin cancer, largely through advertising and marketing campaigns. Despite US Federal Trade Commission actions against the industry, the American Suntanning Association reported in 2015 that it successfully lobbied the US Centers for Disease Prevention and Control (CDC) to remove a disclaimer from its website linking sunbed use to a 75% increase in melanoma risk [12, 13].

## Harassing scientists: personally targeting, attempting to silence, or diminishing the credibility of scientists responsible for research findings inconvenient to industry

Companies can:

- Accuse scientists of scientific misconduct or attack their credibility;
- Threaten scientists’ career security or financial well-being, sometimes through real or threatened industry lawsuits; or
- Harass scientists by abusing open-records requests or subpoenas.

For example, the agrochemical company Monsanto attempted to discredit the World Health Organization’s International Agency for Research on Cancer (IARC) and its experts, who determined in 2015 that the herbicide glyphosate was a probable carcinogen. Fearing that the findings would spur stricter regulations globally, Monsanto-targeted independent scientists on the IARC glyphosate workgroup through open-records requests, requested deliberative scientific documents, and worked with members of US Congress to threaten funding cuts from the US to IARC [14, 15].

## Buying credibility: using scientific credibility of academic institutions to push corporate agendas while leveraging funding to secure support from the scientific community

To gain public legitimacy and distract from harmful practices or products, industry may fund science that advances their public profile, or develop and finance academic partnerships, chairmanships, and research positions.

For example, Purdue Pharma, makers of Oxycontin, launched in the US, the Massachusetts General Hospital Purdue Pain Program. Purdue staff reported that the program would give the company “name recognition among medical students, residents, and the public, as well as political protection against the efforts to address the opioid crisis.” A 2018 Massachusetts Attorney General lawsuit alleged that Purdue started the program to gain access to doctors and residency trainings because it “would help Purdue sell more opioids in Massachusetts [16].”

## Manipulating government officials: inappropriately influencing policymakers to undermine the role of independent science in policy

**Fig. 2:**
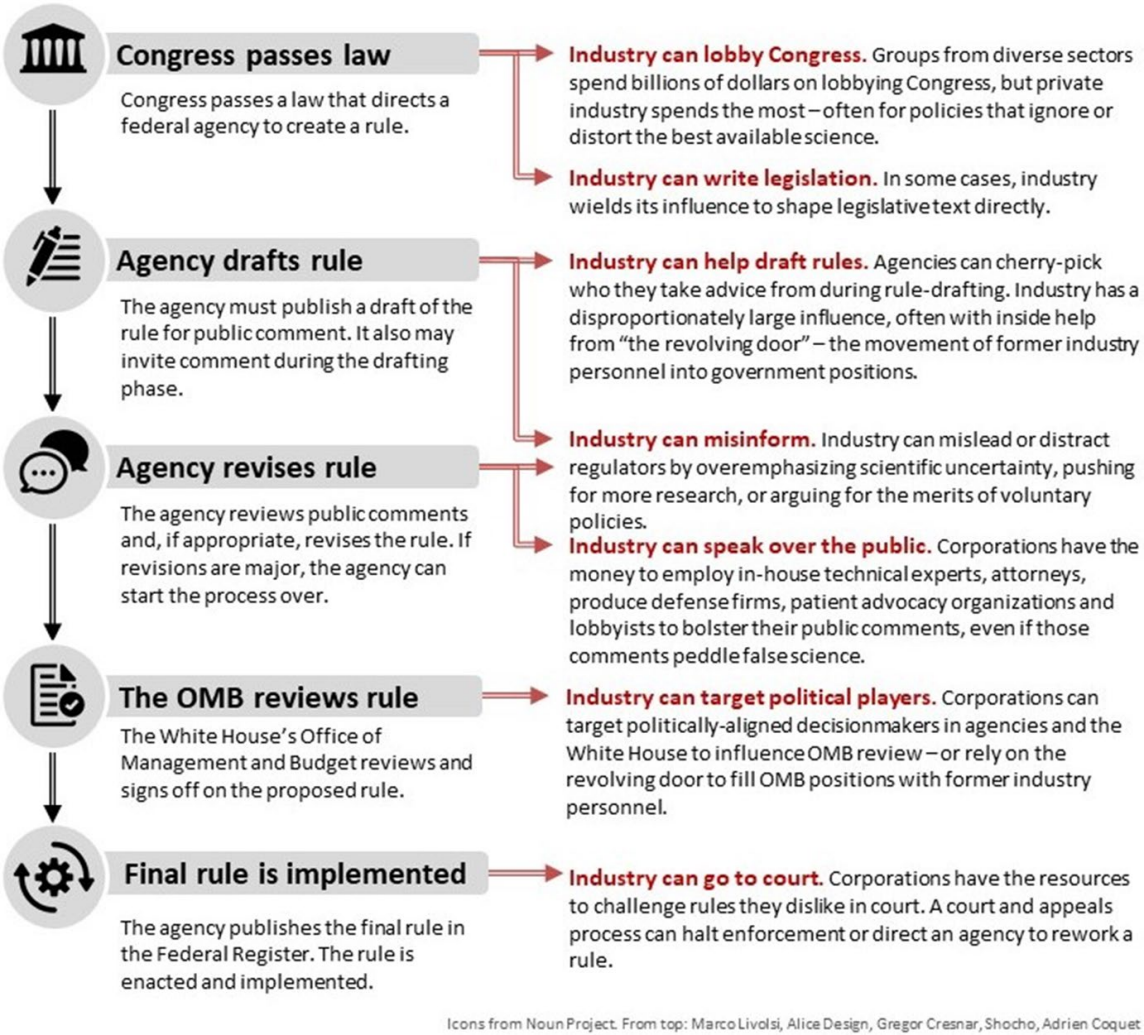
The federal science-based rulemaking process and industry’s tactics to influence it. Industry may interfere with science-based decisions made by US government officials in the executive and legislative branches throughout the federal policymaking process

In 2010, the Supreme Court case *Citizens United v. FEC* overturned campaign finance restrictions, thereby empowering corporations to spend unlimited amounts on US elections [17]. Industries routinely wield their financial power to influence elected officials to co-craft industry-friendly policies, exploit gaps or weaknesses in regulatory schema, or stymie unfavorable regulations, in addition to setting regulatory agencies’ priorities or stocking agencies with former industry personnel (Fig. 2).

For example, in 2009, the tobacco and electronic cigarette industry began lobbying against attempts to regulate flavored e-cigarettes, despite strong evidence that they increased the likelihood of addiction among children. A key industry lobbyist in the US was Andrew Perraut, a former Office of Management and Budget (OMB) analyst who later became a policy director at JUUL Labs, an e-cigarette company. After a series of meetings Perraut had with OMB about the rule, the White House blocked FDA’s efforts to ban e-cigarettes. Although the FDA issued a ban on flavored e-cigarettes in 2019, it exempted liquids for tank-style products and menthol flavorings as a result of industry pressure—exemptions that experts believe put children at continued risk [18].

## Discussion

Corporate actors have a role to play in policy, but their outsized influence on public health decision making must shift, and government actors must be held accountable for permitting playbook strategies [2, 3]. This shift can only be catalyzed by a system that includes incentives for upholding scientific integrity and firewalls that enforce principles of scientific integrity, promote transparency in policymaking, protect independent science, and punish behavior that undermines science for the public good (Table 1). These mechanisms must be consistently applied, enforced, audited, and—upon detecting new loopholes—updated.

**Table 1:** Firewalls to protect scientific integrity

Strategy	Target	Firewall	Outcome of firewall	Firewall case studies
Faking Science	- Journals - University scientists - Federal/state/local government	- Peer review - Journal disclosure of funding sources - Policies to manage COIs - Database of registered clinical trials - Separation of industry funding from product safety testing	- Prohibiting the publication of studies with flawed methodologies - Identify and reject/prevent papers with clear financial objectives - Greater transparency of funding sources and COIs, with penalties (such as retraction) for concealment	Most journals now require COI disclosure of published authors, but only a small percent have enforcement mechanisms. *Environmental Health Perspectives’* policy results in a three-year ban from publishing in the journal and retraction for inaccuracies in reporting COIs, when warranted [22]
Harassing Scientists	- University scientists - Federal scientists - Advisory committee members	- Whistleblower protection laws - Institutional legal support for scientists	- Scientists able to speak out about topics inconvenient for industry without fear of reprisal - Scientists able to obtain legal support and injunctive relief if harassment occurs	The Climate Science Legal Defense Fund [31] provides legal services for scientists harassed for doing their work. It was founded to protect legal aid to climate scientist Dr. Michael Mann during an ideology-driven lawsuit by a fossil fuel funded group [32]
Manufacturing Uncertainty	- Journals - Media - Congress - Public	- Peer review - Journalistic COI standards - Database of registered clinical trials - Trade association disclosure of membership and relationships - Regulatory comment periods require COI disclosure - Campaigns that expose front groups that circulate patently false information	- Greater transparency of funding sources and COIs - Separation of scientific debates and critiques and voiced by legitimate scientific sources from critiques by groups that have a financial stake in manufacturing doubt in science - Penalties for presenting fake information as scientific consensus	The Health Effects Institute serves as a boundary organization, providing an effective conduit between the auto industry and the EPA when researching policy-related questions on air pollution health impacts research [33]
Buying Credibility	- Universities - Scientific associations - Patient advocacy organizations - NGOs	- Enforcing directives from IRBs and scientific integrity boards - Barring research contracts that have strings attached - Robust COI management - Barring certain industry funding at universities	- Clear and strong enforcement mechanisms to enhance scientific integrity and research protocols - Divestment from industries that have proven themselves to be bad actors	The University of California system divested from fossil fuel funding in September 2019 [34]. In 2018, seventeen public health school deans, including Harvard and Johns Hopkins, refused to take any money from the SmokeFree World Foundation funded by the Philip Morris tobacco company [35]
Manipulating Government Officials	- Federal/state/local government - Advisory Committees - Congress	- Fully implemented and enforced scientific integrity policies at science agencies - Strengthening COI policies to prevent “revolving door” between industry and regulatory agencies (a minimum five-year “cooling-off” period between these jobs) - Mandated independent, external science advisory committees - Increased oversight by Congress on COIs and ethics agreements of political officials and advisory committee members	- Less “regulatory capture” at government agencies by industry - Increased ability and willingness to regulate industries when the science demonstrates public health harm - Policies are more grounded in robust scientific evidence - Creation of a more independent regulatory culture -Disincentives and accountability mechanisms when undue influence occurs	In 2010, the White House Office of Science and Technology Policy directed federal agencies to develop scientific integrity policies which protect the use, development, and communication of science at federal agencies. More than 24 federal agencies have since developed scientific integrity policies, though the policies vary greatly in strength, scope, completeness, and effectiveness [25, 36]

To discourage conflicts of interest (COIs) in published research, journals, scientific societies, and academic institutions can develop and enforce strong scientific integrity and disclosure policies governing author, editor, and reviewer conflicts and funding sources. Requiring separation of industry funding and the research evaluating a product’s safety or harms is invaluable [19]. Many journals follow conflict disclosure guidelines established by the Committee on Publication Ethics and the International Committee of Journal and Medical Editors [20]. If journals discover undisclosed COIs, they can impose temporary bans on authorship or issue corrections, retractions, or letters of concern [21]. For transparency around sponsorship of clinical trials, registration on a publicly accessible database like ClinicalTrials.gov is a good start, but more must be done so researchers, journal editors, and the public can understand financial conflicts and hold researchers accountable [22].

To curb funding abuses that potentially endanger research integrity, institutions can establish firewalls between industry funders and researchers. Third-party intermediaries, like independent government agencies, can receive industry money and reallocate it to vetted researchers or organizations for conducting product testing. Some academic institutions have created systems to prevent commercial interests from unduly influencing research, including committees to manage COIs and enforcement mechanisms for ethics agreements [23]. Outside the US, some governments have launched initiatives to separate industry funding from product safety testing. For example, the Italian Medicines Agency taxes the pharmaceutical industry’s drug promotion to fund research on drug efficacy and safety [19].

To ensure the independence and integrity of science in policymaking, strong procedural firewalls can discourage inappropriate affiliations between scientific advice, stakeholder and public input, and political decisions. If real or perceived conflicts could threaten scientific integrity, decisionmakers can be recused from involvement. Candidates for political appointments, advisory committees, and other positions should be vetted, and those with direct ties to regulated industries should be excluded from consideration for regulatory roles. The US Office of Government and Ethics, which registers and tracks COIs, should also have the resources to conduct adequate monitoring and the power to work with agencies to penalize appointees for undisclosed conflicts or breaches in ethics agreements [24]. Additionally, federal agencies could require corporations to compete for regulatory compliance by setting standards for independent review of company-submitted data that affects public or environmental health. Unlike reviews by privately hired compliance entities with financial interest in giving favorable reports, peer reviews by independent organizations incentivizes transparency and accountability.

To ensure transparency of stakeholders' conflicts, stronger accountability requirements can reduce undue industry regulatory influence. Regulatory agencies’ visitor logs, meeting materials, and communications with stakeholders on policy issues can be made public. Further, groups or individuals who publicly comment, or otherwise contribute published scientific studies or unpublished data related to the health consequences of products or practices during the rulemaking phase can be required to disclose interests [25]. For example, the US Department of Labor requested that public commenters disclose funding sources for a 2013 rule to set workplace standards for silica [26]. And in 2017, the French government fined a scientist €50,000 for testifying on the costs of air pollution without disclosing that he was funded by an oil company [27].

## Conclusion

The scientific community faces frightening realities: the public’s ability to distinguish science from pseudoscience is declining [28], corporate efforts to influence evidence-based policymaking are intensifying [29], and foundational processes to protect science—including the integrity and independence of peer review systems and advisory committees—are under attack [30]. The examples covered here offer a glimpse into the problems that are ubiquitous worldwide, and the solutions presented can be adopted outside of the United States. All stakeholders, in and outside the global scientific community, must develop and enforce policies that ensure transparency and accountability in science-based decision making. Stopping use of the disinformation playbook and protecting science as a tool for the collective good will renew the public’s faith that government decision making is working to protect human health, rather than corporate profits [30].
